# Pain Ratings, Psychological Functioning and Quantitative EEG in a Controlled Study of Chronic Back Pain Patients

**DOI:** 10.1371/journal.pone.0031138

**Published:** 2012-03-14

**Authors:** Stefan Schmidt, José Raúl Naranjo, Christina Brenneisen, Julian Gundlach, Claudia Schultz, Holger Kaube, Thilo Hinterberger, Daniel Jeanmonod

**Affiliations:** 1 Department of Environmental Health Sciences, University Medical Center, Freiburg, Germany; 2 Institute of Transcultural Health Studies, European University Viadrina, Frankfurt/Oder, Germany; 3 Department of Psychosomatic Medicine and Psychotherapy, University Medical Center, Freiburg, Germany; 4 Bernstein Center for Computational Neuroscience, University of Freiburg, Freiburg, Germany; 5 Interdisciplinary Pain Unit, University Medical Center, Freiburg, Germany; 6 Center for Functional Neurosurgery, Solothurn, Switzerland; 7 Brain, Mind and Healing Program, Samueli Institute, Alexandria, Virginia, United States of America; 8 Department of Psychosomatic Medicine, University Medical Center, Regensburg, Germany; University Hospital La Paz, Spain

## Abstract

**Objectives:**

Several recent studies report the presence of a specific EEG pattern named Thalamocortical Dysrhythmia (TCD) in patients with severe chronic neurogenic pain. This is of major interest since so far no neuroscientific indicator of chronic pain could be identified. We investigated whether a TCD-like pattern could be found in patients with moderate chronic back pain, and we compared patients with neuropathic and non-neuropathic pain components. We furthermore assessed the presence of psychopathology and the degree of psychological functioning and examined whether the strength of the TCD-related EEG markers is correlated with psychological symptoms and pain ratings.

**Design:**

Controlled clinical trial with age and sex matched healthy controls.

**Methods:**

Spontaneous EEG was recorded in 37 back pain patients and 37 healthy controls.

**Results:**

We were not able to observe a statistically significant TCD effect in the EEG data of the whole patient group, but a subsample of patients with evidence for root damage showed a trend in this direction. Pain patients showed markedly increased psychopathology. In addition, patients' ratings of pain intensity within the last 1 to 12 months showed strong correlations with EEG power, while psychopathology was correlated to the peak frequency.

**Conclusion:**

Out of several possible interpretations the most likely conclusion is that only patients with severe pain as well as root lesions with consecutive thalamic deafferentation develop the typical TCD pattern. Our primary method of defining ‘neuropathic pain’ could not reliably determine if such a deafferentation was present. Nevertheless the analysis of a specific subsample as well as correlations between pain ratings, psychopathology and EEG power and peak frequency give some support to the TCD concept.

**Trial Registration:**

ClinicalTrials.gov NCT00744575

## Introduction

Chronic pain is one of the most frequent chronic diseases. The largest subgroup of chronic pain conditions is lower back pain which is often considered as the one condition causing the largest financial damage to the economy in terms of treatment and work days lost [1]. The life time prevalence for chronic back pain in Germany is estimated to be 24% for men and 30% for women [2]. Accordingly there are many interdisciplinary efforts to investigate the genesis and maintenance of chronic pain. While there is growing knowledge on the peripheral and spinal neuronal mechanism of pain chronification processes there is only limited understanding of central nervous changes in chronic pain [3].

Considerable information on the central neural mechanisms of chronic severe neurogenic (or neuropathic) pain has been obtained from studies on specific thalamocortical patterns [4]–[10]. According to this approach there is a relationship between neurogenic pain and thalamocortical dysfunctional rhythmic activity. This pattern originates in the presence, due to thalamic deafferentation, of low threshold calcium spikes (LTS) with a mean interburst discharge rate of 4 Hz at the limit between the delta and theta ranges. These bursts could be directly measured using single unit recordings in the thalamus of patients with neurogenic pain [11]–[14]. It was furthermore demonstrated, using quantitative EEG analyses, that this dysfunctional pattern results in an increased and shifted mode of cortical processing, providing thus a non-invasive neural marker of chronic pain [5], [10]. This overly accentuated neural rhythmicity was termed Thalamocortical Dysrhythmia (TCD) [5], [15]. In 2008 Sarnthein and Jeanmonod [8] published a paper where they report about simultaneous recording of local field potentials in the medial thalamus and surface EEG in 10 patients during surgery. Patients showed the presence of a theta peak in the EEG spectrum as well as a theta peak in the local field potential spectrum with a maximum coherence in this frequency range.

In order to describe the specific EEG pattern of this TCD Sarnthein et al. [7] compared the EEG spectral activity during rest of 15 patients with severe neurogenic pain with 15 matched healthy controls. Patients showed two marked differences in their power spectra. Overall power was consistently higher in patients over the whole frequency range, mainly in the theta and beta domains. Furthermore their peak of the power spectral density was significantly shifted toward lower frequencies. Patients underwent neurosurgery with a therapeutic lesion in the medial thalamus. From a subgroup of seven patients postoperative EEG was recorded at 3 and 12 months after surgery. All but one patient reported strong pain relief (median 95%) and showed a reduced power compared to before surgery. The power spectrum after 12 months was very similar to that of healthy controls and also exhibited an increased frequency of the peak power. In a similar study by Stern et al. [10] the low frequency EEG overactivity could be localized in cortical regions associated with pain processing [see also 16].

Based on these findings it can be assumed that the presence of TCD can be detected in the surface EEG and that this pattern (i.e. increased overall power, power peak decreased towards theta range) may be a marker of severe chronic neuropathic pain. Since TCD seems to be related to the perpetuation and chronification of pain we wondered if this specific EEG pattern could also be found in a wider population of chronic pain patients.

We were furthermore interested in psychological correlates of chronic back pain and whether these correlates were also related to the EEG patterns under consideration. It is well known that chronic pain and especially chronic back pain is associated with psychological distress [17]–[19] in general and depression in particular [20]–[22]. This is most likely due to a self-maintaining process of pain and illness behaviour, less activity and social withdrawal which then results in psychological distress and has a negative effect on pain experience.

In the current investigation we replicated the study of Sarnthein et al. [7] on a different, i.e. more general sample [23]. Thereby we had the following for objectives: (i) to assess whether a TCD-related EEG pattern generalizes to patients with only moderate chronic pain and (ii) also to patients with chronic pain which is not due to a neuropathic origin while all other methodological aspect were duplicated from [7]. In order to have a more clearly defined sample of chronic pain patients than in the predecessor studies we restricted our sample to chronic back pain patients and compared them with a sample sex and aged matched healthy controls. We furthermore assessed (iii) psychopathology-related symptoms and psychological functioning in order to evaluate psychological impairment in relation to chronic back pain. Additionally we aimed to assess (iv) whether EEG power and peak frequency is related to psychological symptoms as well as pain ratings.

## Methods

### 1. Design

We performed a controlled clinical trial with age and sex matched healthy persons as controls. The EEG spectral density as well as a set of questionnaires assessing psychological symptoms and psychological functioning of a sample of 37 patients with chronic back pain was compared with a sample of 37 healthy sex and age matched controls. Data within the patient group were split into patient with neuropathic pain vs. non neuropathic pain and compared to each other. The protocol for this trial and supporting CONSORT checklist are available as supporting information; see Checklist S1 and Protocol S1.

### 2. Patients

Inclusion criteria for our study were (1) chronic back pain of least 1 year, (2) daily complaints about back pain, (3) an average rating of at least 5 for the average pain of the last 12 months on a VAS ranging from 0 = no pain at all to 10 = worst pain possible (4) age 18 to 70 years and (5) command of German language. Exclusion criteria were (1) the presence of psychiatric conditions including substance dependence, (2) immune suppressive treatment, (3) life threatening diseases, (4) participation in other clinical trials. Patients were recruited between July 2008 and December 2008 by public announcements and via pain specialists, neurologists and orthopaedists as well as through the Interdisciplinary Pain Unit of the University Medical Center. Patients underwent telephone screening before being invited to an intake interview to the Department of Environmental Health Sciences were all measurements took place. They were examined by an MD to determine inclusion and exclusion criteria before being included in the trial. Patients were offered participation in a behavioural intervention free of costs after EEG measurement.

Healthy controls were recruited via public announcements. They had to be healthy and were not allowed to suffer from any pain. Controls were remunerated with 20 Euros for their participation in the EEG recording.

The trial was approved by the University Medical Center ethic's committee (please see Supporting Information for full letter) and was registered before start of recruitment at www.clinicaltrials.gov NCT00744575. All patients and controls gave written informed consent.

#### 2.1 Sub samples

The sample of chronic pain patients was furthermore divided into two sub-samples. This was done by an MD with special training in chronic pain management. Patients were classified as either having pain of neuropathic origin (or not). Assessment took place according to the definition published by Treede et al. [24] which was also taken over by the International Association for the Study of Pain (IASP). They define neuropathic pain as “Pain arising as a direct consequence of a lesion or disease affecting the somatosensory system” [24, p. 1630].

#### Sample Size

Sample size was determined on the basis of the predecessor study, which found significant results with N = 15 patients. Since we wanted to split our sample in two subsamples we recruited with N = 37 more than twice as many patients in order to arrive at least at the same statistical power for each subsample than in the original study.

### 3. Measurements

Questionnaires

Patient filled in the following questionnaires:

1. *EuroQol Quality of Life Questionnaire EQ-5D* [25]. This is a simple 5 item questionnaire for health related quality of life (HRQoL). In addition there is a VAS regarding the general health state.
2. *Brief Symptom Inventory BSI* [26], [27]. This is the 53 items short form of the SCL 90-R a symptom check list functioning as screening instrument for psychopathology.
3. *Hospital Anxiety and Depression Scale HADS* [28], [29] a 14 items screening instrument for anxiety and depression disorders.
4. *Pain Perception Scale PPS* [30] A questionnaire measuring pain perception with the two subscales sensory and affective pain, with 14 and 10 items, respectively.
5. *Chronic Pain Grade CPG* [31]. This scale assesses the severity of chronic pain problems. It provides a set of several visual analogue scales (VAS) assessing pain intensity during the last three months. We added modified versions of this VAS in order to also assess average pain during the last 4 weeks and during the last 12 months.
6. *Questions on Life Satisfaction QLS* [32]. This is a 32 item German questionnaire assessing generic as well as health related life satisfaction on 8 different dimensions.
7. The *general intake form* of the Interdisciplinary Pain Unit. This is an 18 page intake questionnaire especially designed for pain patients which collects information on sociodemographics, medication, pain localisation and prior treatments.

Healthy control participants had only to fill in BSI, EQ-5D, QLS, and HADS.

### 4. EEG recording

EEG was recorded (bandpass filtered 0–200 Hz; A/D rate 1000 Hz) with 72 channel amplifier (Quickamp, MES, Munich, Germany) according to the international 10/20 system, from 60 electrode sites distributed throughout the whole head of the participants. The ActiCap System (MES, Munich, Germany), that includes a cap with active electrodes was used. Diagonal EOG was recorded bipolarly from above and below the right eye to exclude trials contaminated with eye movements from further analysis. The ground electrode was placed in the left mastoid and the output EEG data was average referenced.

Electrode impedances were kept under 5 kΩ. All measurements took place between 8 and 12 am in order to avoid sleepiness. All participants had to abstain from caffeine on the day of measurement since caffeine is known to influence theta activity [33]. EEG assessment took place in a sound and electromagnetically attenuated chamber in the neurophysiology lab of the department. During the experimental session, we recorded 5 minutes of EEG with eyes closed and 5 minutes with open eyes while the participant was sitting on a chair in a sound and electromagnetically shielded, dimly lit chamber. Only the data of the eyes closed condition was used for the subsequent analyses.

### 5. Data processing

All data analyes were performed with the commercial software Brain Vision Analyser 2.0 (MES, Munich, Germany) and custom scripts in MatLab (MathWorks, USA). Artefacts due to ocular movement were first eliminated applying the Gratton & Coles algorithm, as implemented in Brain Vision Analyzer. Remaining EEG segments contaminated with eye movements, EMG or other artefacts of technical origin were rejected off-line by visual inspection. EEG channels with residual artefacts (mainly peripheral sites in the temporal and frontal regions) were excluded from further analysis. Data were filtered with a bandpass filter (Butterworth Zero Phase Filter 1–30 Hz, slope 48 dB/oct) and segmented into 4 seconds epochs (with 2 s overlapping), allowing for a frequency resolution of 0.25 Hz. For each participant, 100 free-of artefact segments were included in further analyses. At this point two different analyses were conducted in parallel: In the first one, a discrete 4000 sample Fast Fourier Transformation (FFT) was computed for each segment (conceptual replication of Sarntheim et al., [7]. In the second analysis, previous to the FFT calculation, EEG epochs were first transformed into the reference-free current source density distribution (CSD), which reflects the underlying cortical activity and removes nearly all volume conduction effects [34]. The CSD algorithm was estimated using the spherical spline interpolation method [35] as implemented in BrainVision Analyzer 2.0. These two different procedures will be further referred as *replication* and *CSD analysis*. In both cases, topographic distribution of power spectral density (PSD) was obtained by averaging across all 100 epochs. Given that PSD from all channels were similar, we averaged the log-transformed spectra across all channels for each participant. Grand-average power spectral density was then computed across subjects for comparing different sub-groups (i.e. patients vs. controls, neuropathic pain vs. non-neuropathic etc.).

### 6. Data Analysis and Statistics

Similar to the study of Sarntheim et al. [7], the frequency of the dominant peak (*peak frequency*) and the log-transformed PSD values at this frequency (*peak power*) was determined for each participant, in order to assess differences between the subgroups. Since for some participants the power spectral density did not show only one clearly dominating peak but looked broadly distributed within the frequency range of interest (4–12 Hz), we defined two other indices to grasp the overall EEG activity and its dominant frequency within this frequency range. First, the mean peak frequency and according standard deviation were computed across all participants. Next the lower and upper limits of a frequency-based ROI were defined by ± two standard deviations of the mean peak frequency. This resulted in two ROIs of 7.34 to 12.43 Hz for the replication and 7.07 to 12.43 Hz for the CSD analysis respectively. Additionally for each participant and type of analysis, the *overall power* was computed as the log-transformed sum of all PSD values within the empirically defined ROIs. In addition, we calculated for each ROI its *centre of gravity* CoG, which is the frequency at which the whole EEG power is split into two equal parts. This CoG splits the ROI into two parts with the same overall power. By this procedure we arrived at four different indices (*peak frequency, peak power, centre of gravity and overall power*) which were then passed on to the subsequent statistical analysis.

All statistics were calculated with SPSS for Windows 15.0 (SPSS Inc, Chicago, Il). We tested all variables comparing between patients and controls for normal distribution by the Kolmogorv Smirnoff Test. If they proved to be normally distributed we applied the t-test for independent samples otherwise we used the non parametric Mann-Whitney U-Test. For the correlation of questionnaire and pain rating data with EEG parameters we applied the non parametric Spearman's rho. As an effect sizes measure for group differences *Cohen's d* was applied [36].

## Results

### 1. Participants

**Figure 1:**
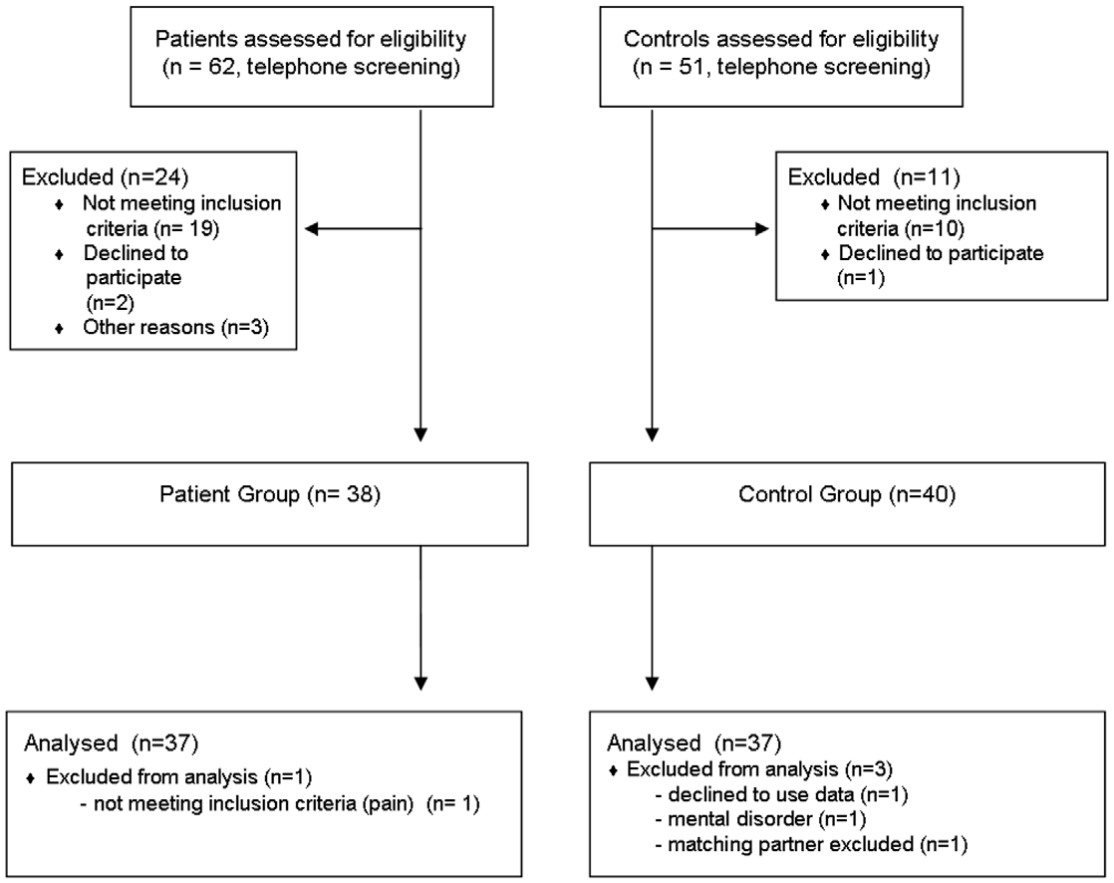
CONSORT flow chart on patient recruitment.

On the basis of telephone screenings 38 patients were invited for intake examination and were included as well as and 40 healthy controls of same age (±3 yrs) and sex. Eighteen patients were classified as having neuropathic pain and 19 as non neuropathic. One patient and 3 control persons had to be excluded from the analysis (see figure 1 for flow chart on recruitment). Basic sample characteristics can be seen from table 1. An overview on diagnoses, pain duration and classification as well as medication for patients only can be found in table S1 (supporting informations).

**Table 1:** Sociodemographic data of the patients and controls.

	Patients	Controls
N	37	37
Age (SD)	50.0 (10.21)	49.8 (10.82)
Sex (m/f)	9/28	9/28
Education Level		
9 years	6	1
11 years/GCSE	15	9
A-level/college entry level	15	26
missing	1	1
Family Status		
married	15	13
married, living sep.	0	1
widow	1	0
divorced	7	7
Single	13	14
missing	1	1

### 2. Questionnaire Data

#### 2.1 HrQoL, Life Satisfaction and Psychological Symptoms

Questionnaires on HrQoL and life satisfaction as well as the two screening scales regarding psychological symptoms were filled in by both groups. In table 2 health related parameters of the two samples are compared and tested for significant differences.

**Table 2:** Means, standard deviations, significance and effect size Cohen's *d* for health related parameters comparing back pain patient with healthy controls.

	Patients	Controls	p	Effect size d
HrQoL	N = 36^c^	N = 37		
EQ 5D VAS scale	54.8 (19.8)	84.0 (11.0)	<.001^a^	1.82
Symptom List BSI	N = 36^c^	N = 37		
GSI	0.67 (0.51)	0.29 (0.33)	<.001^a^	0.88
somatization	0.85^d^ (0.66)	0.34 (0.41)	<.001^a^	0.93
obsessive-comp.	1.05 (0.69)	0.50 (0.40)	.001^a^	0.98
depression	0.66 (0.72)	0.20 (0.36)	.001^a^	0.81
anxiety	0.68 (0.62)	0.29 (0.45)	.001^a^	0.72
phobic anxiety	0.51 (0.75)	0.14 (0.26)	.002^a^	0.66
interpersonal sensitivity	0.78 (0.81)	0.42 (0.49)	.02^b^	0.54
hostility	0.53 (0.48)	0.29 (0.46)	.04^b^	0.51
paranoid ideation	0.59 (0.70)	0.33 (0.49)	.08^b^	0.43
psychoticism	0.34 (0.46)	0.19 (0.23)	.08^b^	0.41
Life Satisfaction QLS	N = 37	N = 37		
generic	29.8^c^ (36.4)	53.3 (31.6)	.008^a^	0.69
health related	26.5 (34.4)	74.7 (33.2)	<.001^a^	1.43
HADS	N = 37	N = 37		
anxiety	7.92 (4.16)	3.68 (3.00)	<.001^a^	1.17
depression	7.54 (4.36)	2.52 (2.42)	<.001^a^	1.42

a Mann-Whitney-U-Test,

b t-Test for independent samples.

c N = 36, one patient excluded due to too many missing items.

d N = 34.

Patients and controls showed marked differences in all psychological variables. For thirteen out of fifteen subscales these were significant. Effect sizes range from medium to very large.

#### 2.2 Pain Specific Questionnaire Measures

Pain specific questionnaires measures were filled in by the patient group only (N = 37). We assessed pain ratings by VAS (0 to 10) and pain perception by the Pain Perception Scale. As can be seen in table 3 the neuropathic and non-neuropathic subgroups showed significant differences regarding their pain perception but reported pain intensities in the same range.

**Table 3:** Means and standard deviations of pain specific questionnaires in the sample chronic back pain patients, and the two sub samples “neuropathic pain” and “non neuropathic pain”.

	All patients (N = 37^a^)	neuropathic (N = 18^a^)	non neuropathic (N = 19^a^)	Sign. p
PPS				
affective	33.2 (10.06)N = 35	36.7 (10.34)	29.5 (8.56)N = 17	.03
sensory	19.6 (6.29)N = 36	22.1 (6.40)	17.2 (5.28)N = 18	.02
Pain VAS				
present moment	4.46 (2.19)	4.89 (2.22)	4.05 (2.15)	.25
av. last four weeks	5.72 (2.07)	5.83 (2.21)	5.63 (1.98)	.77
av. last three months	5.35 (1.62)	5.33 (1.78)	5.37 (1.50)	.95
average twelve months	5.61 (1.82)N = 36	5.86 (1.78)	5.36 (1.89)N = 18	.42

P-values reflect a comparison of the means of the sub-samples by a t-test for independent samples.

a if not stated otherwise.

### 3. EEG Results

**Figure 2:**
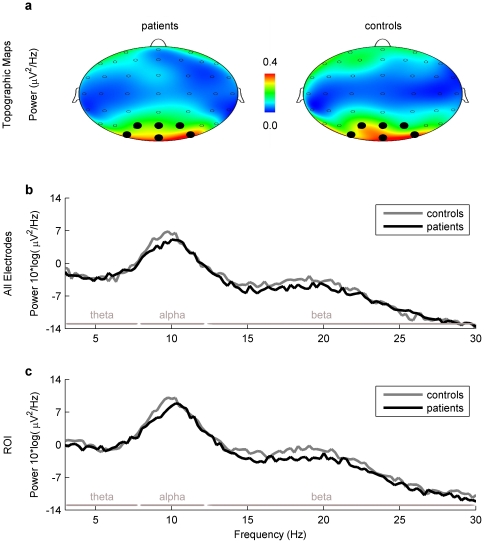
Topographic maps (top view) and average power spectra. The topographical distribution of EEG power (2a) had a maximum at parieto-occipital electrodes for the dominant power peak (9.5 Hz) in both patient and control groups. Note that differences in mean power spectrum are minimal between patient (black) and control group (grey) across all electrodes (2b) and within the ROI (2c) for all the frequency ranges theta, alpha and beta (delimited by horizontal grey bars). The ROI included electrodes Oz, O1, O2, Pz, P1, P2 and POz, PO3, PO4. In this top view only electrodes Pz, P1, P2 and POz, PO3, PO4 (marked with black spots) are observed.

Figure 2 shows a descriptive display of the spontaneous EEG activity measured over all electrodes (2b) in the two groups with reference to the respective power bands (theta, alpha, beta) as well as a topographic display of the power distribution (2a).

#### 3.1 Predefined analyses

For the comparison between patients and controls we used as described in the *Methods* section two different frequency indices, i.e. *Peak Frequency* and *Center of Gravity (CoG)*. Furthermore we assessed the specific power at the peak frequency (*Peak Power*) as well as within the defined ROI (*Overall Power*). Analyses of EEG spectral data were based on *surface EEG* values (conceptual replication of [7] ) as well as based on the underlying *CSD* activity. Thus we arrived at eight relevant indices. Complete data for the comparison of patients with controls can be found in table 4.

**Table 4:** Means and standard deviations for the four main EEG indices and for two analyses methods for patients and matched controls.

	mean (SD)		Sign. p	d
	patients(N = 37)	controls(N = 37)		
Surface EEG				
Peak Freq (replication) [Hz]	10.07 (1.06)	9.64 (1.06)	.09	0.41
Peak Power (replication) [µV^2^/Hz]*	10.68 (6.38)	11.53 (5.62)	.55	−0.14
CoG [Hz]	9.86 (.49)	9.72 (.49)	.24	0.29
Overall Power [µV^2^/Hz]*	17.82 (5.24)	18.82 (4.86)	.40	−0.20
CSD				
Peak Freq. [Hz]	10.11 (1.18)	9.78 (.89)	.18	0.32
Peak Power [µV^2^/Hz]*	28.53 (6.26)	28.82 (4.93)	.83	−0.05
CoG [Hz]	9.89 (.47)	9.88 (.41)	.88	0.02
Overall Power [µV^2^/Hz]*	36.15 (5.16)	36.33 (4.06)	.59	−0.12

P-values reflect a comparison of the means of the sub-samples by a t-test for independent samples. ‘replication’ indicates variables which were also applied in the predecessor study.

* = log-transformed.

**Figure 3:**
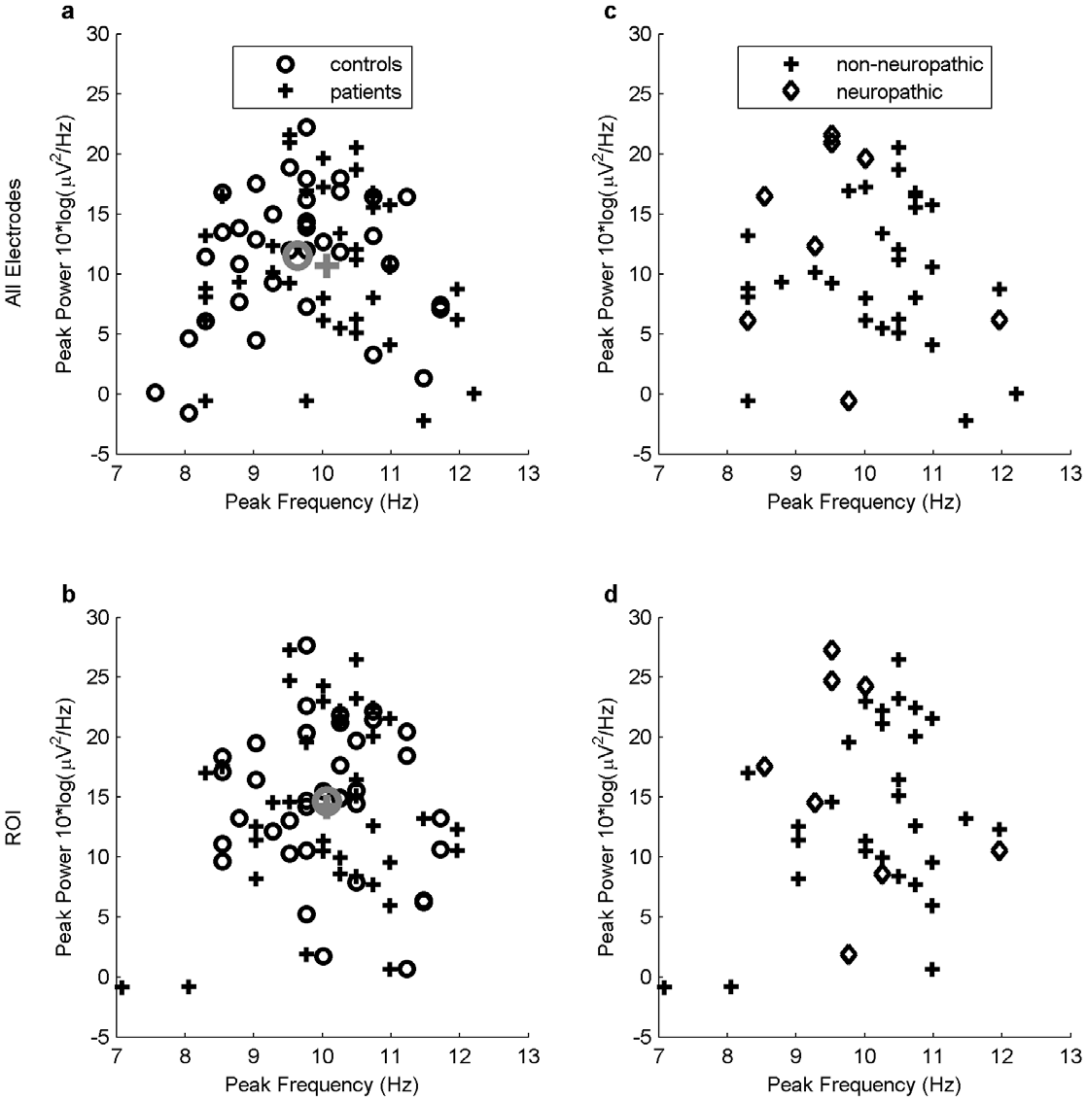
Scatterplot of EEG indices *Peak Power* and *Peak Frequency* (Replication) for individual participants. Left panel (*replication*): Values for patient (crosses) and control groups (circles) across all electrodes (3a) and within the ROI (3b). Mean values in the patient and control groups are shown in grey color. Right panel: Mean values for neuropathic patients fulfilling the necessary conditions for root lesion (diamonds) and non-neuropathic patients (crosses) across all electrodes (3c) and within the ROI (3d). The ROI included electrodes Oz, O1, O2, Pz, P1, P2 and POz, PO3, PO4.

As can be seen in table 4 and figure 3a unlike in the Sarnthein et al. study no difference could be found between the patient and the control group regarding their peak power and peak frequency. Similarly, our analysis based on additional indices did not yield any significant difference between patients and controls. Differences between groups were very small and in opposite to the expected direction.

The topographical power distribution in figure 2a clearly shows the strongest power is in the occipital region. In order to perform a more specific analysis we defined a region of interest (ROI) for parieto-occipital area. The ROI consisted of nine electrodes (Oz, O1, O2, Pz, P1, P2, POz, PO3, PO4). The resulting power density distribution for this ROI only can be seen in figure 2c, the combined chart depicting Power and Peak Frequency simultaneously in figure 3b. Obviously there was no difference between the all electrodes and the ROI approach.

We repeated the same analysis for the two subgroups of neuropathic and non-neuropathic patients. Both samples were compared with their respective controls for significant differences in the eight EEG indices. We did not find any significant results in these comparisons. The p-values for the comparison of neuropathic patients with controls ranged from *p* = .29 to .83, for non neuropathic patients from *p* = .10 to .95 respectively.

#### 3.2 Post-hoc analyses

In order to find out whether our failure to replicate the findings by Sarnthein was due to the extension of our sample to patients with moderate chronic pain we selected a subsample with severe pain from all patients. Fourteen patients reporting a pain intensity ≥7 were included in this sub-sample, 8 out of them with neuropathic and 6 with non neuropathic pain. In this subsample six of the eight indices (all four CSD indices as well as surface EEG *Overall Power* and *Peak Power*) showed differences in the expected direction but none of these differences reached significance, p-values ranged from *p* = .23 to .69). The according effect sized for these differences ranged from *d* = 0.15 to 0.47.

Another possibility why our replication failed may be the fact that only a limited number of patients in our sample fulfilled the necessary conditions for a root lesion resulting in TCD. Thus, one of us (JD) assessed patients' records for the following criteria: (a) ICD diagnosis either M51.2 (Other specified intervertebral disc displacement: Lumbago due to displacement of intervertebral disc) or M54.4 (Lumbago with sciatica), and (b) irradiation of the pain and/or somatosensory deficits in the lower extremity. The goal was here to ascertain more tightly the presence of neuropathic pain mechanisms, i.e. of a root damage as the source of thalamic deafferentation. Eight patients fulfilled these criteria and were compared to their respective controls in an exploratory post-hoc analysis. None of the eight EEG indicators yielded a significant difference (p-values ranging from .09 to .87), but contrary to the whole sample, patients in this subsample had higher power values than their respective controls. The effect sizes for the four power indices were in the range from d = 0.20 to d = 0.44. The findings for the frequency parameters were inconsistent with two times patients having lower frequencies and two times controls. These 8 patients tended, however, to cluster in the top left part of Figure 3c and 3d, speaking for a correlation trend with higher EEG power and lower peak frequency.

### 4. Correlations with EEG parameters

In order to see whether there is a relationship between subjective questionnaire data and EEG parameters we calculated the respective correlation coefficients (see table 5). We report only those questionnaire scales for which significant correlations were found. Additionally table 6 shows correlation coefficients between EEG parameters and all pain ratings.

**Table 5:** Correlation coefficients between EEG parameters and questionnaire data.

	BSI obs.-comp.	QLS generic	QLS health	PPS affective
Frequency Indices				
Replic. CoG	−.04	.13	.24	−.09
Replic. Peak Freq.	−.30	.44**	.42*	−.36*
CSD CoG	−.23	.28	.38*	−.19
CSD Peak Freq.	−.45**	.39*	.48**	−.36*
Power Indices				
Repl. Overall Power	.25	−.16	−.20	.22
Repl. Peak Power	.20	−.17	−.21	.16
CSD Overall Power	.22	−.14	−.15	.16
CSD Peak Power	.20	−.18	−.20	.19

Only scales with significant correlations are reported.

*p<.05,

**p<.01. In the BSI and PPS higher values indicate more symptoms or stronger pain perception. In the QLS higher values indicate larger life satisfaction.

**Table 6:** Correlation coefficients between EEG parameters and pain ratings.

	present moment	av. last four weeks	av. last three months	av. last year
Frequency Indices				
Replic. CoG	−.06	.09	−.06	−.10
Replic. Peak Freq.	−.21	−.07	−.20	−.21
CSD CoG	−.15	−.08	−.18	−.17
CSD Peak Freq.	−.29	−.25	−.29	−.32
Power Indices				
Repl. Overall Power	.22	.48**	.40*	.44**
Repl. Peak Power	.22	.47**	.39*	.40*
CSD Overall Power	.36*	.50**	.41*	.46**
CSD Peak Power	.32	.47**	.43**	.45**

Only scales with significant correlations are reported.

*p<.05,

**p<.01.

All significant correlations found between EEG parameters and psychological variables were only with frequency indices. All significant correlations were in the same direction associating larger psychopathology with lower frequency values or better psychological functioning with higher frequency respectively. On the other hand all significant correlations between pain ratings and EEG were only with power indices. Here higher pain ratings correlated consistently positive with EEG power values. Highest correlations were found for averaged pain ratings of the last four weeks, three months and 12 months but not for pain at the moment of EEG recording.

## Discussion

In our comparison of subjective and objective data of chronic back pain patients with sex and age matched controls we see most clearly strong differences in psychopathology and psychological functioning between the two groups. Patients scored significantly worse regarding psychological symptoms in general (BSI, Global Severity Index) as well as on seven of the nine specific subscales with the other two approaching significance. Effect sizes ranged from 0.41 to 0.98 and are all medium or large in size. In the HADS questionnaire, a screening tool for depression and anxiety, the effect sizes were even higher with 1.17 (anxiety) and 1.42 (depression). This finding was supported by the more generic measures of HrQoL and life satisfaction. Here the differences were still larger with the topmost value being close to two standard deviations (1.82, HrQoL). It is evident form our findings that the continuous back pain present in these patients is accompanied by a severe reduction in quality of life, life satisfaction as well as increased psychopathology compared to a healthy control group. This is in line with findings of other investigators, e.g. for psychological distress measured by the SCL 90-R (which is the longer version of the BSI scale applied here) in relation to chronic back pain [17]–[19].

Regarding their pain intensity patients reported medium to strong values. The inclusion criteria requested an average pain rating during the last months of at least 5 on a VAS from 0 to 10. On the day of the measurement patients showed a rather moderate rating with a mean of 4.5. Average pain ratings during the last four weeks, last three months and last twelve months were all between 5 and 6. We split our sample into the two subgroups of 18 pain patients with neuropathic origin and 19 patients with non-neuropathic origin. These subgroups showed no differences regarding their pain intensity but differences regarding their pain perception. Neuropathic patients reported more intense pain perceptions in the sensory and in the affective domain. This makes sense on the background that neuropathic pain can also be diagnosed on the basis of pain sensations which are markedly different on several sensory dimensions [37]. In our case the diagnosis was based on a definition also adopted by the IASP [24] and the differences in pain perception give support to this procedure. This definition however does not separate damages of small nerve branches in different tissues like capsules, ligaments or muscles from root damage, which is the only damage able to give rise to the ischialgic neuropathic deafferentation syndrome, typically seen after “failed back surgery”. As shown by a post-hoc subsample analysis, only 8 patients fulfilled criteria for an at least probable root lesion, indicating that 10 patients were selected wrongly as being neuropathic by the above-mentioned definition.

Regarding the EEG results we were not able to replicate the findings by [7]. The whole patient group neither showed a significant increase in power nor a slowed dominant frequency. This was true for our conceptual replication as well as for our EEG analysis based on computation of the current source density (CSD), which has the advantage of reflecting the underlying cortical activity. For both analysis methods as well as for two ways of calculating the relevant power or frequency variables we found only very slight non-significant differences which were all in the opposite direction of the original findings. However, when the sample was restricted to the fourteen most severe pain patients the indices pointed in the expected direction but did not reach significance. And when the sample was restricted to the eight neuropathic patients selected as having root damage and thus susceptible to elicit TCD, all power indices pointed into the expected direction.

How can these only slight effects be explained? There are several possibilities we can think of.

### 1. Small sample

It might be that our sample was too small to detect significant differences in the relevant EEG parameters. While this cannot be ruled out, we do not consider this interpretation as very likely. We had 37 patients and 37 controls compared to 15 patients and 15 controls in the forerunner study. However it might be interesting to note that Sarnthein et al. did not present results of inference statistics on comparing the means of frequency and power variables as we did. Rather they performed a discrimination analysis where they could show that on the basis of the two variables *peak frequency* and *peak height* 87% of all participants could be correctly classified as patients or controls. The fact that the power spectra of patients and controls showed clear differences can also be seen from the graphical display in Sarnthein et al. (2006, fig. 1 & 2). In contrast the same graph of our data (fig. 2) shows no differentiation of the two groups at all.

### 2. Back pain does not result in TCD

Another interpretation is that back pain is due to a different type of mechanism than the pain types reported in the predecessor study. Out of the 15 patients in the Sarnthein et al. study none suffered from back pain. Pain locations were mainly trigeminal and leg. Back pain is due to nociceptive (or somatogenic) mechanisms elicited by increased nociceptor activation in peripheral tissues. This is indeed the opposite of a neuropathic dynamic induced by reduced thalamic activation (or deafferentation) and causing the EEG pattern described as TCD. This study provides an important negative result: pain syndromes due to nociceptive mechanisms do not give rise to TCD, or possibly only to thalamocortical overactivities too discrete to show up with the applied techniques.

### 3. Only very strong pain elicits statistically significant EEG overactivities, i.e. a significant TCD

Another difference in comparison to the forerunner study was that patients in the Sarnthein et al. study had much stronger pain, which lead them to surgical treatment. Mean pain intensity was rated 6.9 (*SD* = 1.18, *median* = 6.5, no time interval reported) compared to 5.7 (*SD* = 2.07, *median* = 5.0, mean pain intensity during the last four weeks) in our study. This difference may be even a bit masked due to the pain medications many patients took in both studies. In the Sarnthein study 10 out of 15 patients (67%) took either benzodiazepines, antiepileptics, antidepressants, opiates or a combination of them; in our study the rate for the same medications was only 43%. To test for this hypothesis we drew a subsample of 14 patients with a pain rating of ≥7 (average over the last four weeks). We still did not find any significant differences compared to healthy controls but now the direction of the effect changed for 6 out of 8 EEG variables into the hypothesized direction with small to medium effect sizes. This is at least a hint pointing into the direction of a TCD pattern.

### 4. An initial, TCD-inducing event (root lesion) does not guarantee the long term maintenance of TCD

In our study, only 8 patients suffered from pain syndromes compatible with root damage and thus fulfilling the criteria of Sarnthein et al. The modes of selection in the two studies were very different: in Sarnthein et al., patients were specifically selected for root damage as the source of chronic therapy-resistant neuropathic syndromes and at the exclusion of dominant nociceptive situations, which were either absent or at best coupled to the neuropathic component as a factor of secondary relevance. In this context, the possibility was excluded that the neuropathic pain had become secondary or even irrelevant as time passed, replaced by nociceptive or psychogenic components. In our study, there exists indeed the possibility that an initial neuropathic dynamics, due to root lesion and causing TCD at the onset of pain, decreased/disappeared over time, leaving the place for and the lead to a chronification of their back pain by one or both of these components. Interestingly, the selected subsample of eight patients with most likely root lesion and thus TCD showed, similar to the subsample of patients with stronger pain, small differences for their power indices in the expected direction (larger power in patients) which was not true for the full sample. In addition, these patients tended to cluster in the top left part of Figure 3c and 3d, correlating with higher EEG power and lower peak frequency. However, one has to keep in mind that this is an exploratory post-hoc analysis.

According to the last three interpretations, it can be assumed that a majority of the patients in our study had either not severe enough and/or non-neuropathic pain components. Because of this, they could not elicit as a group a statistically significant TCD pattern.

We found medium size but highly significant correlations between the individual pain rating and the EEG parameters as well as between the subjective variables and the EEG patterns. Regarding pain intensity we only found significant correlations with power indices but not with frequency indices. In contrast questionnaire data correlated only significantly with frequency indices but not with power related variables.

The correlations with pain intensity were strongest for average pain intensity during the last 1 to 12 months but were weaker and mostly not significant with pain intensity during the day of measurement. This finding clearly indicates that there is a relationship with continued pain experience and EEG power. Since the correlation was smallest for the pain present during the measurement it can be assumed that the increase in power is not due to actual pain experience but due to a chronification process due to persistent pain as this is hypothesized in TCD. The correlation coefficients of *r* = .39–.50 demonstrate that 15% to 25% of the variance in EEG power is due to pain experience in the last year. This finding indicates that severe chronic pain in a group of patients having predominantly nociceptive and/or psychogenic components results in larger EEG power. To explain this finding, we have evidence [38] that psychological factors can indeed be at the source of a TCD development, which is localized bilaterally over large prefrontal paralimbic and associative cortical areas.

Interestingly and in the same direction, the shift of the peak frequency was correlated with some of the self-reported data. Here all significant correlations also pointed into the expected direction showing a shift towards smaller frequency with an increase in psychopathology or a reduction in life satisfaction. These findings are quite likely not due to chance. For the whole set of EEG frequency variables correlated with subjective indices we can expect by chance 3.4 significant correlations (*α* = .05, four EEG indices, 17 subscales) while we find seven. Here it is also interesting that we do find correlations mainly for life satisfaction (QLZ) but not for strength of psychopathological symptoms as e.g. reflected by GSI. Additionally there is a significant correlation with affective pain perception. In order to assess if these results could also be explained by the fact that chronic pain is correlated with poorer life satisfaction and larger psychopathology as reflected in table 2 we tried to regress EEG variables by both pain ratings and psychological variables in some exploratory analyses. None of these models could explain more than what was already reflected in the correlation tables. Pain ratings could not explain additional variance in frequency parameters and psychological variables did not enter regression models for power indices (data not reported).

In conclusion we were not able to generalize the findings of a TCD-related, statistically significant EEG pattern to patients with moderate back pain. The number of patients suffering from an ongoing and dominant neuropathic dynamics was too small to replicate the significant data from Sarnthein et al. Nevertheless three of our findings are in accordance with the TCD concept. This is the fact that the EEG patterns in our patient group are more in accordance with TCD predictions in patients with severe pain ratings than in patients with moderate pain, as well as in patients with thalamic deafferentation and ongoing dominant neuropathic dynamics. Moreover there is a strong correlation between pain intensity during the last 1 to 12 months and EEG power but not with present pain. Thus the EEG parameters chosen on the basis of the TCD mechanism do indeed reflect a relationship with strong and also persistent pain experience. While our findings on one hand support the TCD concept, they also demonstrate that these specific changes in the EEG cannot be used as a marker for chronic pain as a whole, and that a distinction, which is anyway of primary clinical relevance, must be done between neuropathic, nociceptive and psychogenic mechanisms. However, whether EEG-based TCD-analyses are of use for any of these subgroups was beyond the scope of our study.

## Supporting Information

Checklist S1 CONSORT checklist.
(DOC)

Protocol S1 Trial Protocol.
(PDF)

Table S1 Clinical description of all patients.
(DOC)
