# Supplementary material for: Pain Ratings, Psychological Functioning and Quantitative EEG in a Controlled Study of Chronic Back Pain Patients
Source: PLoS One. 2012 Mar 14;7(3):e31138. doi: 10.1371/journal.pone.0031138 (PMC3303776; doi:10.1371/journal.pone.0031138)
Supplement: Protocol S1 — Trial Protocol. (PDF) [file pone.0031138.s002.pdf]

Studienprotokoll zur Vorlage bei der Ethikkommission

Identifikation einer neurophysiologischen Schmerzsignatur bei chronischen Rückenschmerzen und deren Veränderung durch eine verhaltensmedizinische Intervention – eine Pilotstudie

Antragsteller

Dr. Stefan Schmidt, PD Dr. Thilo Hinterberger, Dipl. Phys. José Raul Naranjo, cand. med. Ann-Mareike Lemme

Institut für Umweltmedizin und Krankenhaushygiene, Sektion Komplementärmedizinische Evaluationsforschung.

Prof. Dr. Holger Kaube, Dr. Marianne Lüking

Interdisziplinäres Schmerzzentrums, Neurozentrum, Universitätsklinikum Freiburg

Abstract

In drei neueren Studien konnte gezeigt werden, dass chronischer neuropathischer Schmerz im Spontan-EEG mit einer allgemeinen Amplitudenerhöhung, einer Überaktivierung im theta-Frequenzbereich und einer Verschiebung der dominanten Frequenz in einen niedrigeren Bereich einhergeht. Dieses Muster wird als thalamocortikale Dysrhythmie bezeichnet, die im Nucleus centralis lateralis des Thalamus generiert wird und durch die thalamocortikale Rückkopplung zu den spezifischen EEG-Veränderungen in der kortikalen Schmerzmatrix führt. Zunächst sollen diese EEG-Befunde einer Schmerzsignatur bei Patienten mit chronischen Rückenschmerzen nach schmerzauslösendem neuropathologischen Erstereignis repliziert werden. Im nächsten Schritt sollen auch Patienten mit chronischem unspezifischem Rückenschmerz auf eine Dysrhythmie im Spontan-EEG überprüft werden. Ziel ist es, den Zusammenhang zwischen subjektivem Schmerzempfinden und EEG-Signatur in einem Patientenkollektiv mit unterschiedlichster Schmerzanamnese explorativ zu untersuchen, um die Bedeutung und Differentialdiagnostik der berichteten EEG-Signatur besser zu verstehen. Weiterhin sollen die Patienten mit einem innovativen kognitiven achtsamkeitsbasierten Gruppenverfahren (Mindfulness based stress reduction MBSR) behandelt werden. Zur Identifikation des EEG-Musters werden die Patienten mit einer gesunden, gematchten Stichprobe verglichen. 24 Patienten, die dieses EEG-Muster aufweisen, werden dann in einem Pilottrail (feasibility trail) einen 8-wöchigen MBSR-Kurs absolvieren. Messzeitpunkte sind zu Baseline, am Ende der Intervention und nach weiteren 10 Monaten. Neben dem EEG werden Schmerz, Schmerzakzeptanz, Lebensqualität, psychische Belastung, Achtsamkeit und Lebenszufriedenheit als weitere Zielgrößen gemessen.

Stand der Forschung

Chronische Rückenschmerzen sind Alltag vieler Menschen und verursachen von allen Krankheitsbildern mit die höchsten Gesundheitskosten. Die Lebenszeitprävalenzen liegen bei 24% (Männer) und 30% (Frauen) (Neuhauser, Ellert & Ziese, 2005). Die Mechanismen zur Entstehung, Zunahme, Aufrechterhaltung und Verselbstständigung chronischer Schmerzen sind Gegenstand vielfältiger interdisziplinärer Forschung und bislang erst zum Teil gut verstanden.
Als zentralnervöses Korrelat chronischen Schmerzes wurde die sogenannte kortikale Schmerzmatrix identifiziert. Dazu gehören Gebiete des primären und sekundären somatosensorischen Kortex, die Insel Region, der vordere Kortexanteil des Gyrus cinguli und Thalamuskernbereiche (2000).
Die Ergebnisse von drei aktuellen Studien einer schweizerischen Neurochirurgengruppe legen dar, dass bei chronischen Schmerzpatienten mit neuropathischen Schmerzen im Spontan-EEG ein spezifisches Muster (‚Schmerzsignatur') festzustellen ist. Es besteht aus einer generellen Amplitudenerhöhung, einer Überaktivierung im theta-Frequenzbereich sowie einer Verschiebung der dominanten Frequenz in einen niedriegeren Bereich. Dieses Muster, das als thalamokortikale Dysrhythmie bezeichnet wird, entsteht im Ncl. centralis lateralis des Thalamus und führt durch die thalamokortikale Rückkopplung zu dem beschriebenen spezifischen EEG-Muster (Sarnthein, Stern, Aufenberg, Rousson & Jeanmonod, 2006; Stern, Jeanmonod & Sarnthein, 2006). Bei der Lokaliserung zeigte sich dass das Muster in den oben benannten relevanten Bereichen der Schmerzmatrix aufzufinden ist. Es ist hier erstmals einer Forschergruppe gelungen, eine spezifische *dynamische* Signatur chronischen Schmerzes im Gehirn zu identifizieren. Durch eine gezielte therapeutische Läsion am Ncl. centralis lateralis gelingt es diese Rückkopplungsschleife zu unterbrechen, was zu einer sehr starken Schmerzreduktion (Median 95%) auch bei Patienten mit Rückenschmerzen führte. Die thalamokortikale Dysrhythmie reduzierte sich 12 Monate nach der Operation signifikant (Stern, Jeanmonod & Sarnthein, 2006).
Die Autoren untersuchten bisher ausschließlich Patienten, deren chronische Schmerzerkrankung sich auf ein eindeutiges neuropathologisches Erstereignis in der Schmerzanamnese zurückführen ließ (neuropathischer Schmerz). Jedoch besteht die berechtigte Vermutung, dass sich dieses Muster in einer ähnlichen Form auch bei Patienten findet, bei denen die Erstmanifestation des Schmerzes laut Anamnese eher nicht neuropathologisch bedingt (chronischer unspezifischer Schmerz) ist (Jeanmonod, 2007). In der vorliegenden Arbeit sollen sowohl Patienten mit chronischen unspezifischen Rückenschmerzen als auch mit chronischen Rückenschmerzen nach schmerzauslösendem neuropathischen Erstereignis in die Studie aufgenommen und diesbezüglich verglichen werden. Interessant ist weiterhin, ob eine Verbesserung der Schmerzen, wie sie durch eine verhaltensmedizinische, nicht-invasive und nicht-pharmakologische Intervention erreicht werden kann, ebenfalls zu einer Reduktion der thalamokortikalen Dysrhythmie führt.
Eine der innovativsten verhaltensmedizinischen Interventionen ist das auf Achtsamkeit und Akezptanz basierende kognitive Gruppenprogramm „Mindfulness-based stress reduction (MBSR)", das von Jon Kabat-Zinn (1982) entwickelt wurde. Achtsamkeit wird psychologisch charakterisiert als ein Zustand der Aufmerksamkeit, in dem jeder einzelne Moment bewusst und nicht-wertend wahrgenommen wird. Die Übung der Achtsamkeit ist dazu geeignet, vorherrschende Reaktionsmuster zu unterbrechen, bestehende Bewertungen und Attributionen zu überprüfen und durch eine Verantwortungsübernahme für die eigene Situation neue gesundheitsfördernde Verhaltensweisen zu entwickeln. Die Wirksamkeit der MBSR-Intervention bei chronischen Erkrankungen im Allgemeinen konnte von uns in einer Metaanalyse nachgewiesen werden (Grossman, Niemann, Schmidt & Walach, 2004). Zahlreiche Studien weisen auch auf die spezifische Wirksamkeit bei chronischen Schmerzen hin (Grossman, Tiefenthaler-Gilmer, Raysz & Kesper, 2007; Kabat-Zinn, Lipworth & Burney, 1985; Kingston, Chadwick, Meron & Skinner, 2007; Plews-Ogan, Owens, Goodman, Wolfe & Schorling, 2005; Sephton et al., 2007).

In einer Pilotstudie wurde MBSR bei älteren Patienten mit chronischen Rückenschmerzen untersucht. Es zeigte sich unter anderem eine klinisch signifikante Verbesserung auf der physischen Funktionsskala des generischen Lebensqualitätsfragebogen SF-36 (d=0.46 im Vergleich zur Kontrollgruppe), die sich im Laufe des Follow-ups noch verbesserte (Morone, Greco & Weiner, 2008).
Es kann daher davon ausgegangen werden, dass chronifizierte Schmerzsyndrome auf diese verhaltensmedizinische Intervention ansprechen und zu einer Veränderung der Schmerzwahrnehmung (Intensität, Qualität) selbst als auch der Lebensqualität (physische, psychische, emotionale, soziale Funktion) durch die MBSR-Intervention möglich sind und durch eine De-Chronifizierung auch zu einer Veränderung der thalamokortikalen Dysrhythmie im Spontan-EEG führen können.

Ziele

Unsere Studie gliedert sich in zwei Teile. Im ersten Teil soll eine heterogene Population von Patienten mit chronischen Rückenschmerzen auf die Anwesenheit einer spezifischen Schmerzsignatur im EEG untersucht werden.
Im zweiten Teil sollen Patienten, die diese Signatur aufweisen einer verhaltensmedizinischen Intervention unterzogen werden, die besonders darauf abzielt die chronifizierenden und schmerzaufrechterhaltenden Aspekte des Schmerzsyndroms zu verändern. Die Wirksamkeit dieser Evaluation bei dieser Population soll hinsichtlich neurophysiologischen und subjektiven Zielkriterien evaluiert werden.

Im Einzelnen verfolgen wir folgende Ziele:

- Eine Replikation des von Sarnthein et al. (2006) berichteten Befundes einer thalamokortikalen Dysrhythmie (TCD) in der Schmerzmatrix bei Patienten mit chronischen neuropathisch bedingten Rückenschmerzen im Vergleich zu einer gesunden Kontrollgruppe.
    - Der chronische Rückenschmerz wird als ‚neuropathisch bedingt' charakterisiert, wenn ein eindeutiges neuropathologisches Erstereignis in der Schmerzanamnese aufzufinden ist.
- Untersuchung einer Population von Patienten mit chronischen unspezifischen Rückenschmerzen auf die Anwesendheit einer TCD im Vergleich zu einer gesunden Kontrollgruppe.
    - Der chronische Rückenschmerz wird als unspezifisch charakterisiert, wenn kein eindeutiges neuropathologisches Erstereignis in der Schmerzanamnese aufzufinden ist.
- Evaluation einer verhaltensmedizinischen Intervention (achtsamkeitsbasierte Stressbewältigung) bei Patienten mit chronischen Rückenschmerzen und einer diagnostizierten TCD hinsichtlich:
    - Der Veränderung der TCD nach 2 und 12 Monaten
    - Der Veränderung der Schmerzwahrnehmung (Intensität, Qualität) nach 2 und 12 Monaten
    - Der Veränderung der allgemeinen Lebensqualität (physische, psychische, emotionale, soziale Funktion) nach 2 und 12 Monaten
    - Der Veränderung der Schmerzakzeptanz und der Achtsamkeit nach 2 und 12 Monaten
- Untersuchung des Zusammenhangs zwischen Veränderungen der TCD und den subjektiv berichteten Maßen nach 2 und 12 Monaten

Arbeitsprogramm

Fragestellungen/ Hypothesen:

1. Lässt sich das EEG-Muster der thalamorkortikalen Dysrhythmie (TCD) in der Schmerzmatrix bei Patienten mit chronischen Rückenschmerzen neuropathischen Ursprungs im Vergleich zu einer gesunden Kontrollgruppe replizieren?
2. Findet sich das EEG-Muster der TCD auch bei Patienten mit chronischen Rückenschmerzen, bei denen sich die Schmerzen auf kein eindeutiges neuropathologisches Erstereignis in der Schmerzanamnese zurückführen lassen (psychogen) im Vergleich zu einer gesunden Kontrollgruppe?
3. Können die EEG-Befunde hinsichtlich TCD bei den beiden Patientenpopulationen Rückenschmerzen nach schmerzauslösendem Erstereignis neuropathischer Genese und chronischen unspezifischen Rückenschmerzen unterschieden werden?
4. Verändert sich das EEG-Muster der TCD durch Behandlung der Patienten mit einem achtwöchigen Streßbewältigungsprogramm (MBSR)?
5. Führt die Behandlung der Patienten mit MBSR zu Veränderungen hinsichtlich berichteter Schmerzwahrnehmung, allgemeiner Lebensqualität, Schmerzakzeptanz, Lebenszufriedenheit und Achtsamkeit.
6. Stehen Veränderung im EEG-Muster der TCD mit Veränderungen in den berichteten Schmerzen, der Schmerzqualität und der allgemeinen Lebensqualität in Beziehung?

Studiendesign:

Es handelt sich um eine diagnostische Studie mit gematchter Kontrollgruppe (cross-sectional study) sowie um eine prospektive Pilot-Studie (feasibility trial).

Intervention:

Bei der Intervention handelt es sich um das Achtsamkeitsbasierte Stressbewältigungstraining (mindfulness based stress reduction, MBSR) nach Jon (1990). Dies ist ein manualisiertes und strukturiertes verhaltensmedizinisches Gruppenprogramm. Die Teilnehmer werden wöchentlich 2,5 Stunden in verschiedenen Meditationstechniken und Yoga von speziell ausgebildeten MBSR-Trainern unterrichtet. Zusätzlich erhalten sie Informationen über Prozesse der Salutogenese. Wesentlicher Bestandteil des Programms sind die täglichen Hausaufgaben von ca. einer Stunde, für die den Teilnehmern Übungsmaterial bereitgestellt wird (CDs, Anleitungen). Zwischen der sechsten und der siebten Kurswoche findet ein Tag der Achtsamkeit statt, bei dem der Transfer der in dem Kurs erlernten Techniken auf den Alltag im Vordergrund steht.

Kontrollen:

Zum Nachweis der thalamokortikalen Dysrhythmie wird eine gematchte Kontrollgruppe gebildet. Für jeden Patienten mit chronischem Rückenschmerz wird eine nach Alter und Geschlecht gematchte gesunde Kontrollperson mit dem gleichen EEG-Paradigma untersucht.

Studienteilnehmer:

Im ersten Teil der Studie (Querschnittstudie) werden Patienten mit chronischem Rückenschmerz auf die Anwesenheit einer thalamokortikalen Dysrhythmie (TCD) untersucht. Dafür gelten die folgenden Ein- und Ausschlusskriterien.

Einschlusskriterien:
- Patienten mit chronischen Rückenschmerzen von mindestens einem Jahr
- Alter 18-70 Jahren
- Gutes deutsches Sprachverständnis

Ausschlusskriterien:
- Vorligen von Psychiatrischen und/oder Suchterkrankungen
- Lebensbedrohliche Krankheit
- Immunsuppression
- Teilnahme an anderen klinischen Studien
- Psychologische und psychiatrische Auffälligkeiten, die den zwischenmenschlichen Kontakt schwer behindern, festzustellen in einem Eingangsinterview

Diese Patienten werden mit einer gesunden, hinsichtlich Alter und Geschlecht gematchten Kontrollgruppe verglichen.

Im zweiten Teil der Studie werden nur Patienten mit chronischen Rückenschmerzen, die im ersten Teil der Studie eine klare TCD aufwiesen, eingeschlossen. Es gelten die folgenden Kriterien:

Einschlusskriterien:
- Patienten mit chronischen Rückenschmerzen von mindestens einem Jahr
- Patienten mit beschriebenem Schmerzmuster im EEG Befund (s. Jeanmonod), sogenannte thalamokortikale Dysrhythmie
- Alter 18-70 Jahren
- Gutes deutsches Sprachverständnis
- Bereitschaft, sich auf die Erfordernisse des Trainings einzulassen
- Motivation und Bereitschaft zur Teilnahme an der Studie; insbesondere Bereitschaft, die studienbedingten Unterlagen sorgfältig und gewissenhaft zu führen

Ausschlusskriterien:
- Vorligen von Psychiatrischen und/oder Suchterkrankungen
- Lebensbedrohliche Krankheit
- Immunsuppression
- Teilnahme an anderen klinischen Studien
- Psychologische und psychiatrische Auffälligkeiten, die den zwischenmenschlichen Kontakt schwer behindern, festzustellen in einem Eingangsinterview

Rekrutierung
Patienten werden aus der Schmerzambulanz des Interdisziplinären Schmerzzentrums am Uniklinikum und von niedergelassenen Schmerzärzten rekrutiert.
Die Eingangsuntersuchung im interdiziplinären Schmerzzentrum umfasst zusätzlich zur ausführlichen Schmerzanamnese die körperliche Untersuchung und die weiterführende Beurteilung

anhand des Schmerzfragebogen der DGSS (Deutsche Gesellschaft zum Studium des Schmerzes; ab Mitte 03/2008 analog der DSF: Deutscher Schmerzfragenbogen (überarbeitete Fassung), des HADS-D (Hospital Anxiety and Depression Scale-Deutsche Version von Herrmann & Buss (1994) und des SF-12 (Kurzform des SF-36, Medical Outcome Study).

Insgesamt sollen 36 Patienten mit chronischen Rückenschmerzen rekrutiert werden. Achtzehn davon mit ‚neuropathisch bedingten' Rückenschmerzen gemäß Definition oben und 18 mit ‚unspezifischen' Rückenschmerzen. Aus dieser Stichprobe sollen 24 Patienten für den zweiten Studienteil, gemäß obenstehender Einschlusskriterien rekrutiert werden. Sollten sich in der Gesamtstichprobe von 36 Patienten nicht genügend Patienten für den zweiten Studienteil finden, werden weitere Patienten rekrutiert.
Weiterhin werden 36 gesunde Kontrollpersonen rekrutiert.

Zielkriterien:

Hauptzielkriterium für die Pilotstudie ist die Veränderung der TCD im Spontan EEG. Nebenzielkriterien sind selbstberichtete Daten zu den Bereichen Schmerzwahrnehmung, allgemeine Lebensqualität, körperliche und psychische Symptome, Lebenszufriedenheit, Schmerzakzeptanz, und Achtsamkeit.

EEG-Messungen
Die EEG-Messungen finden im neueingerichteten neurophysiologischen Labor der Sektion komplementärmedizinische Evaluationsforschung am Institut für Umweltmedizin und Krankenhaushygiene statt.
Das Labor ist mit einem 64-Kanal Verstärker Quickamp (MES, München) mit Aktivelektroden und aktiver Störabschirmung ausgestattet. Dieses System ermöglicht es, ein 64-Kanal EEG in 15-20 Minuten anzulegen und minimiert so den Aufwand für die Patienten um ein Erhebliches. Die Aktivabschirmung des Systems sowie eine akustisch und elektromagnetisch abgeschirmte Messkabine garantieren darüber hinaus eine fehlerfreie Messung.
Die Patienten dürfen vor der EEG-Messung keine koffeinhaltigen Getränke zu sich nehmen. Das Schlafverhalten in der letzten Nacht wird erhoben (Skala VIS-M) und die Patienten werden auf ausreichende Wachheit überprüft, da Schläfrigkeit den Anteil an theta-Frequenzen im EEG ebenfalls erhöhen kann. Es werden zwei Mal 5 Minuten Spontan-EEG im Ruhezustand gemessen, einmal mit geschlossenen und einmal mit offenen Augen.

Fragebogenmessungen
Zu jedem Messzeitpunkt werden folgende Fragebögen erhoben. Die Auswahl der Instrumente folgt den Empfehlungen der IMMPACT Gruppe zu Zielkriterien für klinische Studien mit chronischen Schmerzpatienten. Im Sinne einer Pilotstudie wird eine eher größere Anzahl von Instrumenten eingesetzt, ohne dabei die Patienten jedoch zu überfordern (insgesamt xy Items)

1. Neuropathic Pain Questionnaire (Krause & Backonja, 2003): Der Fragebogen wurde zur allgemeinen Beurteilbarkeit von neuropathischen Schmerzen und zur Differenzierung zu nicht-neuropathischen Schmerzen entwickelt. Er enthält 12 Items, die auf einer Skala von 0 („überhaupt nicht") bis 100 („stärkst möglich") bewertet werden und anschließend mittels eines linearen Modells auf ihre Zugehörigkeit in einer der beiden Gruppen geprüft werden.
2. Schmerzfragebogen (McGill Pain Questionnaire, (Melzack, 1987)): Der Fragenbogen benutzt Adjektivlisten um die Schmerzqualität zu erfassen. Dazu werden die Dimensionen sensorisch, affektiv und evaluativ unterschieden. In der Kurzfassung werden 15 I-

tems von den Patienten auf einer Intensitätsskala von 0=gar keine bis 3=stärkste Ausprägung bewertet. Zusätzlicher Bewertungsteil ist die Visuelle Analogskala für Schmerz (VAS Schmerz): Sie dient der eindimensionalen Ermittlung der Schmerzstärke. Der Patient markiert seine Schmerzstärke auf einer 10 cm langen Linie zwischen dem Anfangspunkt „kein Schmerz“ und dem Endpunkt „stärkster vorstellbarer Schmerz“. Angewandt wird die deutsche Version des McGill Pain Questionnaire: Die Münchener Schmerzwortskala (Mendel & Stein, 1989).

3. EuroQoL-Fragebogen VAS: Aus dem 5 dimensionalen Fragebogen zur Messung der Lebensqualität EQ-5D werden die 5 visuellen Analogskalen verwendet. Sie sind ein schnell anwendbares und sehr gut validiertes Instrument der Euroqol Group (www.euroqol.org) zur subjektiven Beschreibung und Bewertung von Gesundheitszuständen.
4. PLC: Als weitere Skala zur Messung der Lebensqualität wird das *Profil der Lebensqualität chronischer Kranken (PLC)* verwendet. Sechs Subskalen beschreiben die Dimensionen: *Leistungsvermögen* (physischen Handlungsvermögen), *Genuss- und Entspannungsfähigkeiten* (Aspekt des psychischen Vermögens), *Positive Stimmung* (Aspekt des psychischen Befindens), *Negative Stimmung* (Aspekt des psychischen Befindens), *Kontaktvermögen* (Aspekt des sozialen Vermögens) und *Zugehörigkeitsgefühl* (Aspekt des sozialen Befindens).
5. Brief Symptom Inventory Deutsch: Das Brief Symptom Inventory (BSI), eine Kurzform der SCL-90-R, ist ein Instrument zur Erfassung subjektiver Beeinträchtigung durch körperliche und psychische Symptome. Ausgewertet ermöglichen die 53 Items Aussagen zur Symptombelastung, die zu neun Skalen und drei globalen Kennwerten - analog zur SCL-90-R - zusammengefasst werden. Es werden neun Subskalen erfasst: 1. Somatisation, 2. Zwanghaftigkeit, 3. Unsicherheit im Sozialkontext, 4. Depressivität, 5. Ängstlichkeit, 6. Aggressivität/Feindseligkeit, 7. Phobische Angst, 8. Paranoides Denken, 9. Psychotizismus.
6. Schmerzakzeptanz (Pain Acceptance, McCracken, Vowles & Eccleston, 2004): Der Fragebogen (CPAQ-D) liegt seit 2007 in deutscher Übersetzung mit guten psychometrischen Eigenschaften vor und dient als Instrument zur Messung des Akzeptanz-Konstrukts (McCracken, Carson, Eccleston & Keefe, 2004). Die modifizierte Fassung von McCracken, Vowles und Eccleston als sogenannter CPAQ- Fragebogen umfasst 20 Items zur Schmerzakzeptanz. Die jeweilige Aussage soll in einer sechsschrittigen Skala zwischen “trifft niemals” und “trifft immer zu” bewertet werden. Die faktorielle Struktur der deutschen CPAQ-Fassung entspricht weitgehend dem theoretischen Modell.
7. Freiburger Fragebogen zur Achtsamkeit (FFA, Walach, Buchheld, Buttenmüller, Kleinknecht & Schmidt, 2004): Kontextunabhängige Skala zur Selbsteinschätzung der Dimension Achtsamkeit. Der Fragebogen erfasst verschiedene Merkmale von Achtsamkeit wie: Urteilslosigkeit, Akzeptanz, Nicht-Identifikation, Neutralität, Ganzheitlichkeit. Es wird die 14-Item Kurzversion angewendet werden.
8. Fragebogen zur Lebenszufriedenheit (Henrich & Herschbach, 1996): Der Fragebogen umfasst 33 Items zur Lebenszufriedenheit und Lebensqualität. Das Instrument ermöglicht es 8 generelle und 8 gesundheitsspezifische Dimensionen individuell zu gewichten.

Messzeitpunkte

In der Querschnittstudie gibt es nur einen Messzeitpunkt (EEG-Messung). Dieser Messzeitpunkt ist gleichzeitig die Baseline für den zweiten Teil der Studie. Eine weitere Messung findet unmittelbar am Ende der Intervention (nach 2 Monaten) und eine weitere Messung nach 12 Monaten (follow-up) statt.

Studienablauf / Zeitplanung:

0-3	Monate	Vorbereitende Arbeiten (haben bereits begonnen)
1-11	Monate	Rekrutierung der Patienten, Baseline EEG Messungen, Rekrutierung der Kontrollpersonen, Auswertung der EEG Daten hinsichtlich TCD.
8 – 9	Monate	MBSR Kurs 1 (12 Patienten)
9	Monate	Post-Messung Kurs 1
11-12	Monate	MBSR Kurs 2 (12 Patienten)
12	Monate	Post-Messung Kurs 1
20	Monate	12 Monats Follow-up Kurs 1
23	Monate	12 Monats Follow-up Kurs 2
24-25	Monate	Restliche Auswertung und Abschlussbericht (Publikationen)

Datenerfassung und Auswertungsstrategien:
Die EEG Daten werden mit der Software BrainVisionRecorder aufgezeichnet und mit BrainVisionAnalyzer und mit MatLab und der EEGLAB-Suite (http://www.sccn.ucsd.edu/eeglab/) ausgewertet. Die EEG Daten werden in 5 Sekunden Abschnitte aufgeteilt, mit einer Hamming-Funktion gewichtet und mittels Fast Fourier Transformation (FFT) in Power-Spektren überführt. Die Lokalisierungsmodelle werden mit LORETA (low resolution brain electric tomography http://www.unizh.ch/keyinst/NewLORETA/LORETA01.htm) gerechnet. Die Auswertung orientiert sich an der Vorgehensweise von Stern, Jeanmonod & Sarnthein (2006). Fragebogen werden von Hand in MS Access und SPSS for Windows eingeben und mit SPSS ausgewertet.

Statistische Auswertung

EEG-Studie

Zur Unterscheidung von Patienten und Kontrollgruppe wird eine lineare Diskriminanzanalyse ausgeführt. Linearkombinationen der einzelnen EEG-Parameter werden auf ihre Fähigkeit das Verhältnis von Varianz zwischen den Gruppen zu Varianz innerhalb der Gruppen zu maximieren untersucht. Innerhalb der Patientengruppen wird explorativ nach weiteren möglichen dichotomen externen Unterscheidungskriterien gesucht (v.a. neuropathisches Erstereignis vs. kein neuropathisches Erstereignis) und diese in weiteren Diskriminanzanalysen innerhalb der Patientengruppe auf ihre Aussagekraft untersucht.
Für den Vergleich der LORETA Lokalisierung werden basierend auf der kortikalen Schmerzmatrix regions of interest (ROI) gebildet. Für den Gruppenvergleich wird für jede Frequenz zwischen 1-40 Hz (0,5 Hz Schritte) und jedes Voxel im ROI ein t-Wert gerechnet. Es wird für multiples Testen (mehrere Voxel) korrigiert.

Statistische Power

Diese Teil der Studie ist eine Replikation der Arbeiten von Sarntheim et al. (2006) und Stern et al. 2006. In diesen Studien zeigten sich signifikante Unterschiede in den EEG Mustern zwischen 15 bzw. 16 Schmerzpatienten und 15 bzw. 16 Kontrollpersonen, so dass wir davon aus-

gehen bei unseren 36 Patienten und 36 Kontrollpersonen ebenfalls signifikante Unterschiede zu finden. Weiterhin soll die Stichprobe der Patienten in die zwei Untergruppen „ neuropathisch bedingt“ und „unspezifisch“ gruppiert und gegeneinander verglichen werden. Hier gehen wir ebenfalls basierend auf den früheren Publikationen davon aus dass mit N=18 bedeutsame Unterschiede gefunden werden können.

Interventionsstudie
Die Fragebogendaten werden mit einer multivariaten Varianzanalyse mit Messwiederholung für jede Variable separat ausgewertet. Eine Korrektur für multiples Testen findet wegen dem explorativen Pilotcharakter der Studie nicht statt.

Statistische Power
In unser eigenen Metanalyse zeigte sich ein prä-post Effekt für die hier eingesetzte Intervention von $d = 0.50$. Bei 24 Patienten und $d = 0.5$ und eine geschätzten Korrelation von r=.6 zwischen prä und post-Messung haben wir eine Power von $1-\beta = 0{,}77$ (Cohen, 1988).

Literatur

Cohen, J. (1988). *Statistical Power Analysis for the Behavioral Sciences*. Hillsdale: Lawrence Erlbaum.

Grossman, P., Niemann, L., Schmidt, S. & Walach, H. (2004). Mindfulness-based stress reduction and health benefits: A meta-analysis. *Journal of Psychosomatic Research, 57,* 35-43.

Grossman, P., Tiefenthaler-Gilmer, U., Raysz, A. & Kesper, U. (2007). Mindfulness training as an intervention for fibromyalgia: Evidence of postintervention and 3-year follow-up in well-being. *Psychotherapy and Psychosomatics, 76,* 226-233.

Herrmann, C. & Buss, U. (1994). Vorstellung und Validierung einer deutschen Version der "Hospital Anxiety and Depression Scale" (HAD-Skala). *Diagnostica, 40,* 143-154.

Jeanmonod, D. (2007). Der Widerhall von Nervenschädigungen und Schuldgefühlen im Elektroenzephalogramm. In: G. Schönbächler (Hrsg.), *Schmerz. Perspektiven auf eine menschliche Grunderfahrung* (pp. 131-159). Zürich: Chronos.

Kabat-Zinn, J. (1982). An Outpatient Program in Behavioral Medicine for Chronic Pain Patients Based on the Practice of Mindulness Meditation: Theoretical Considerations an Preleminary Results. *General Hospital Psychiatry, 4,* 33-47.

Kabat-Zinn, J. (1990). *Full catastrophe living : using the wisdom of your body and mind to face stress, pain, and illness*. New York, N.Y.: Delacorte Press.

Kabat-Zinn, J., Lipworth, L. & Burney, R. (1985). The Clinical Use of Mindfulness Meditation for the Self-Regulation of Chronic Pain. *Journal of Behavioral Medicine, 8,* 163-190.

Kingston, J., Chadwick, P., Meron, D. & Skinner, T. C. (2007). A pilot randomized control trial investigating the effect of mindfulness practice on pain tolerance, psychological well-being, and physiological activity. *Journal of Psychosomatic Research, 62,* 297-300.

Krause, S. J. & Backonja, M. M. (2003). Development of a neuropathic pain questionnaire. *The Clinical Journal of Pain, 19,* 306-314.

McCracken, L. M., Carson, J. W., Eccleston, C. & Keefe, F. J. (2004). Acceptance and change in the context of chronic pain. *Pain, 109,* 4-7.

McCracken, L. M., Vowles, K. E. & Eccleston, C. (2004). Acceptance of chronic pain: component analysis and a revised assessment method. *Pain, 107,* 159-166.

Melzack, R. (1987). The short-form McGill pain questionnaire. *Pain, 30,* 191-197.

Morone, N., Greco, C. & Weiner, D. (2008). Mindfulness Meditation for the treatment of chronic low back pain in older adults: A randomized controlled pilot study. *Pain, 134,* 310-319.

Peyron, R., Laurent, B. & Garcia-Larrea, L. (2000). Functional imaging of brain responses to pain. A review and meta-analysis. *Neurophysiologie Clinique, 30,* 263-288.

Plews-Ogan, M., Owens, J. E., Goodman, M., Wolfe, P. & Schorling, J. (2005). A pilot study evaluating mindfulness-based stress reduction and massage for the management of chronic pain. *Journal of General Internal Medicine, 20,* 1136-1138.

Sarnthein, J., Stern, J., Aufenberg, C., Rousson, V. & Jeanmonod, D. (2006). Increased EEG power and slowed dominant frequency in patients with neurogenic pain. *Brain, 129,* 55-64.

Sephton, S. E., Salmon, P., Weissbecker, I., Ulmer, C., Floyd, A., Hoover, K. & Studts, J. L. (2007). Mindfulness meditation alleviates depressive symptoms in women with fibromyalgia: results of a randomized clinical trial. *Arthritis & Rheumatism, 57,* 77-85.

Stern, J., Jeanmonod, D. & Sarnthein, J. (2006). Persistent EEG overactivation in the cortical pain matrix of neurogenic pain patients. *Neuroimage, 31,* 721-731.

Walach, H., Buchheld, N., Buttenmüller, V., Kleinknecht, N. & Schmidt, S. (2004). Empirische Erfassung der Achtsamkeit - Die Konstruktion des Freiburger Fragebogens zur Achtsamkeit (FFA) und weitere Validierungsstudien. In: T. Heidenreich & J. Michalak (Hrsg.), *Achtsamkeit und Akzeptanz in der Psychotherapie* (pp. 727-770). Tübingen: DGVT-Verlag.
