# Supplementary material for: Pain Ratings, Psychological Functioning and Quantitative EEG in a Controlled Study of Chronic Back Pain Patients
Source: PLoS One. 2012 Mar 14;7(3):e31138. doi: 10.1371/journal.pone.0031138 (PMC3303776; doi:10.1371/journal.pone.0031138)
Supplement: Table S1 — Clinical description of all patients. (DOC) [file pone.0031138.s003.doc]

Table S1 Clinical description of all patients. Relevant drugs include antiepileptics (AE), antidepressants (AD), opiates (OP), non-steroidal anti-inflammatory drugs (NSAID) and muscle relaxants (MR).

| *No.* | *Age* | *Sex* | *ICD-10* | *diagnosis* | *Pain since yrs* | *Ø pain 4 weeks* | *neuropathic pain (IASP)* | *Drugs* |
| --- | --- | --- | --- | --- | --- | --- | --- | --- |
| 1 | 50 | m | M51.2, M96.1, M54.5 | Postnucleotomy lumboischialgic syndrom | 2 | 5.0 | yes | NSAID |
| 2 | 37 | f | M54.4, M54.5, M48.57, R52.2 | Lumboischialgic syndrome | 9 | 8.0 | yes | NSAID, AD, AE, MR |
| 3 | 23 | f | M54.5 | Lumbalgic syndrome | 10 | 8.0 | no | NSAID,MR |
| 4 | 31 | m | M54.4 | Lumbalgic syndrome | 2 | 5.0 | no | MR |
| 5 | 43 | f | M19.9, R52.2 E10 M54.95 | Thoracic and lumbar pain syndrome | 24 | 7.0 | no | OP |
| 6 | 47 | f | M51.2 | Postnucleotomy lumboischialgic syndrome | 19 | 2.5 | yes | NSAID,MR |
| 7 | 42 | f | M51.2, M54.5, M99.84 | Postnucleotomy lumbalgic syndrome | 9 | 6.0 | yes | NSAID |
| 8 | 47 | m | M41.85, M79.18, M54.6 | Lumboischialgic syndrome | 5 | 6.0 | no | NSAID,OP,AD,AE |
| 9 | 47 | f | M99.84, M43.17 | iliosacral pain syndrome, spondylolisthesis, thoracic pain due to disc protrusion | 3 | 5.0 | no | NSAID,MR |
| 10 | 57 | m | M51.2 | Lumboischialgic syndrome, disc herniation and spondylodesis for spondylolisthesis | 1 | 5.0 | yes | NSAID, OP, AD |
| 11 | 56 | f | M42.14, M62.9, M54.5 | Cervical and lumbar pain syndrome  Osteochondrosis | 7 | 7.0 | no | - |
| 12 | 40 | f | M51.2 | Lumboischialgic syndrome, disc protrusion | 23 | 3.0 | yes | NSAID |
| 13 | 49 | f | M54.5, F33.0 | Lumbalgic syndrome, | 6 | 10.0 | no | NSAID, OP, AD,MR |
| 14 | 63 | f | M51.2, M48.02 | Cervical pain syndrome due spinal canal stenosis, lumbaischialgic pain syndrome due to herniated disc | 42 | 5.0 | yes | NSAID,MR |
| 15 | 57 | f | M51.2, M47.86, M47.84 | Lumbalgic syndrome due to disc protrusion  Spondylarthrosis | 7 | 5.0 | yes | NSAID |
| 16 | 68 | f | M54.5, R52.2 | Lumboischialgic syndrome | 44 | 4.0 | no | NSAID |
| 17 | 48 | f | M48.02, M51.2 | Lumboischialgic syndrome, Cervical spinal stenosis | 4 | 9.0 | yes | NSAID, OP, AD,MR |
| 18 | 54 | f | M51.2, M54.5 | Lumboischialgic syndrome | 21 | 4.0 | yes | NSAID |
| 19 | 51 | f | M19.05, M54.5 | Lumbalgic syndrome | 18 | 5.0 | no | NSAID |
| 20 | 42 | f | M42.1 | Spinal osteochondrosis | 10 | 4.5 | no | NSAID |
| 21 | 57 | f | M51.2, M79.19, F45.4, M54.10 | Lumboischialgic syndrome | 6 | 7.0 | yes | AD,AE |
| 22 | 65 | f | R52.1, M79.10, M47, M54.16, M79.0, M48.02 | Lumboischialgic syndrome | 30 | 8.0 | yes | AE |
| 23 | 57 | m | M47.86, | Lumbalgicsyndrome Spinal osteochondrosis | 11 | 3.0 | no | - |
| 24 | 46 | f | M51.2, M54.4 | Lumboischialgic syndrome | 12 | 2.0 | yes | - |
| 25 | 47 | f | M79.70, M54.4, F45.40 | Fibromyalgia  Lumboischialgic syndrome | 2 | 6.0 | no | NSAID, AE |
| 26 | 49 | f | M51.2, M79.19 | Lumboischialgic syndrome | 14 | 9.0 | yes | NSAID, |
| 27 | 42 | m | M54.14, M54.16 | Lumboischialgic syndrome | 26 | 2.0 | no | OP, AD, |
| 28 | 44 | f | M47.86, M54.5 | Lumboischialgic syndrome | 3 | 4.5 | no | NSAID, OP, AD,MR |
| 29 | 38 | f | M51.2, M54.5 | Lumboischialgic syndrome | 6 | 7.0 | yes | NSAID |
| 30 | 58 | f | M19.05, M79.19 | Lumboischialgic syndrome, spondylodesis for spondylolisthesis, iliosacral pain syndrome | 9 | 5.0 | no | NSAID |
| 31 | 64 | m | M 42.96, M 48.06, M 47.96, M47.82, M 43.16 | Cervical pain syndrome  Postnucleotomy lumboischialgic syndrome | 9 | 4.0 | yes | NSAID |
| 32 | 51 | m | M94.88, M62.98, M54.5, G80.9 | Spinal osteochondrosis  Lumbalgic syndrome | 5 | 8.5 | no | OP |
| 33 | 64 | m | M19.08, M42.16, M99.84 | Spinal osteochondrosis  Lumbalgic syndrome | 55 | 4.0 | no | NSAID |
| 34 | 61 | f | M21.79, M95.5, M54.17, M42.16 | Spinal osteochondrosis  Lumbalgic syndrome | 5 | 5.0 | no | NSAID, OP, AD |
| 35 | 55 | f | M54.5, M47.26 | Lumbalgic syndrome, spondylodesis for  spondylolisthesis | 10 | 7.5 | no | OP, AD |
| 36 | 48 | f | M54.5 | Lumboischialgic syndrome | 13 | 7.5 | yes | NSAID, AD |
| 37 | 43 | f | R52.1, F45.40, M54.5 | Lumboischialgic syndrome | 4 | 8.0 | yes | NSAID, AD |
